# 5-Amino-6-methyl­quinolin-1-ium 3-carb­oxy­propano­ate

**DOI:** 10.1107/S1600536813006673

**Published:** 2013-03-16

**Authors:** Kaliyaperumal Thanigaimani, Nuridayanti Che Khalib, Suhana Arshad, Ibrahim Abdul Razak

**Affiliations:** a School of Physics, Universiti Sains Malaysia, 11800 USM, Penang, Malaysia

## Abstract

The asymmetric unit of the title salt, C_10_H_11_N_2_^+^·C_4_H_5_O_4_^−^, consists of two independent 5-amino-6-methyl­quinolin-1-ium cations and two 3-carb­oxy­propano­ate anions. Both cations are protonated at the pyridine N atoms and are essentially planar, with maximum deviations of 0.026 (3) and 0.016 (2) Å. In the crystal, the cations and anions are linked *via* N—H⋯O and O—H⋯O hydrogen bonds, forming a layer parallel to the *ab* plane. In the layer, weak C—H⋯O hydrogen bonds and π–π stacking inter­actions, with centroid-to-centroid distances of 3.7283 (15) and 3.8467 (15) Å, are observed. The crystal structure also features weak C—H⋯O hydrogen bonds between the layers.

## Related literature

For background to and the biological activity of quinoline derivatives, see: Sasaki *et al.* (1998 ▶); Reux *et al.* (2009 ▶); Morimoto *et al.* (1991 ▶); Markees *et al.* (1970 ▶). For related structures, see: Thanigaimani *et al.* (2013*a* ▶,*b* ▶,*c* ▶); Loh *et al.* (2010 ▶); Sauer *et al.* (2008 ▶). For reference bond-length data, see: Allen *et al.* (1987 ▶). For stability of the temperature controller used for data collection, see: Cosier & Glazer (1986 ▶).
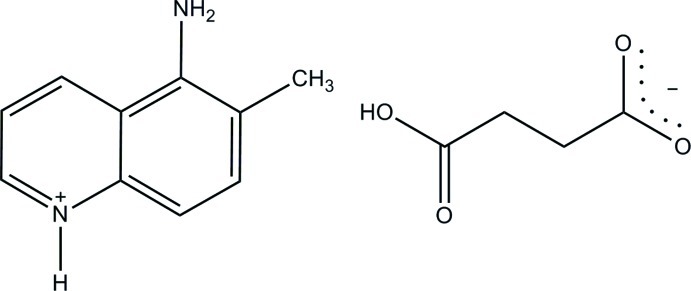


## Experimental

### 

#### Crystal data

C_10_H_11_N_2_^+^·C_4_H_5_O_4_^−^
*M* *_r_* = 276.29
Triclinic, 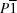

*a* = 8.0784 (3) Å
*b* = 10.8234 (4) Å
*c* = 16.4366 (6) Å
α = 91.608 (2)°
β = 101.039 (2)°
γ = 105.782 (2)°
*V* = 1352.49 (9) Å^3^
*Z* = 4
Mo *K*α radiation
μ = 0.10 mm^−1^
*T* = 100 K
0.31 × 0.17 × 0.16 mm

#### Data collection

Bruker SMART APEXII CCD area-detector diffractometer
Absorption correction: multi-scan (*SADABS*; Bruker, 2009 ▶) *T*_min_ = 0.970, *T*_max_ = 0.984
19941 measured reflections
6733 independent reflections
4669 reflections with *I* > 2σ(*I*)
*R*_int_ = 0.048

#### Refinement

*R*[*F* ^2^ > 2σ(*F* ^2^)] = 0.066
*wR*(*F* ^2^) = 0.200
*S* = 1.04
6733 reflections
395 parameters
2 restraints
H atoms treated by a mixture of independent and constrained refinement
Δρ_max_ = 0.52 e Å^−3^
Δρ_min_ = −0.35 e Å^−3^

### 

Data collection: *APEX2* (Bruker, 2009 ▶); cell refinement: *SAINT* (Bruker, 2009 ▶); data reduction: *SAINT*; program(s) used to solve structure: *SHELXTL* (Sheldrick, 2008 ▶); program(s) used to refine structure: *SHELXTL*; molecular graphics: *SHELXTL*; software used to prepare material for publication: *SHELXTL* and *PLATON* (Spek, 2009 ▶).

## Supplementary Material

Crystal structure: contains datablock(s) global, I. DOI: 10.1107/S1600536813006673/is5252sup1.cif

Structure factors: contains datablock(s) I. DOI: 10.1107/S1600536813006673/is5252Isup2.hkl

Supplementary material file. DOI: 10.1107/S1600536813006673/is5252Isup3.cml

Additional supplementary materials:  crystallographic information; 3D view; checkCIF report

## Figures and Tables

**Table 1:** Hydrogen-bond geometry (Å, °)

*D*—H⋯*A*	*D*—H	H⋯*A*	*D*⋯*A*	*D*—H⋯*A*
N1*A*—H1N*A*⋯O4*A*	0.88 (1)	1.78 (1)	2.667 (3)	177 (4)
N1*B*—H1N*B*⋯O4*B*	0.97 (3)	1.71 (3)	2.664 (3)	170 (3)
O2*A*—H1O*A*⋯O4*A* ^i^	0.83 (2)	1.69 (2)	2.520 (2)	176 (3)
O2*B*—H1O*B*⋯O4*B* ^i^	0.93 (4)	1.60 (4)	2.525 (2)	179 (4)
N2*A*—H2N*A*⋯O3*A* ^ii^	0.99 (5)	1.97 (5)	2.931 (3)	163 (4)
N2*A*—H3N*A*⋯O2*B* ^iii^	0.93 (4)	2.11 (4)	2.937 (3)	149 (3)
N2*B*—H2N*B*⋯O2*A* ^ii^	0.87 (3)	2.22 (3)	3.037 (3)	157 (3)
N2*B*—H3N*B*⋯O3*B* ^ii^	0.87 (3)	2.14 (3)	3.001 (3)	172 (3)
C7*A*—H7*AA*⋯O3*A* ^ii^	0.95	2.42	3.343 (3)	165
C9*A*—H9*AA*⋯O1*A* ^iv^	0.95	2.37	3.271 (3)	158
C7*B*—H7*BA*⋯O3*B* ^ii^	0.95	2.31	3.253 (3)	169
C8*B*—H8*BA*⋯O3*B* ^v^	0.95	2.51	3.323 (3)	143
C9*B*—H9*BA*⋯O4*B* ^v^	0.95	2.52	3.388 (3)	153

Symmetry codes: (i) 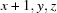; (ii) 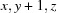; (iii) 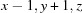; (iv) 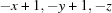; (v) 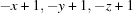.

**Fig. 1.**
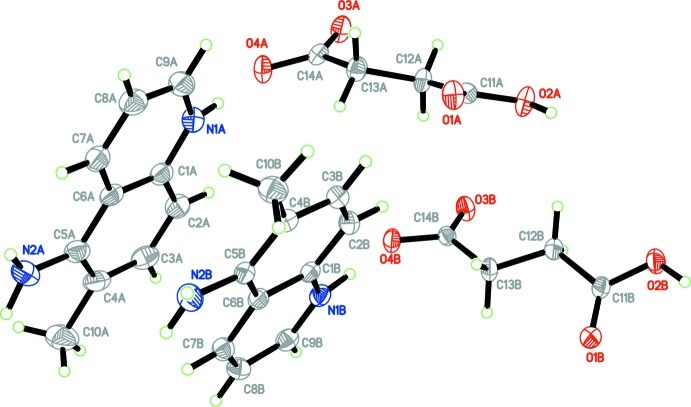
The asymmetric unit of the title compound with atom labels with 50% probability displacement ellipsoids.

**Fig. 2.**
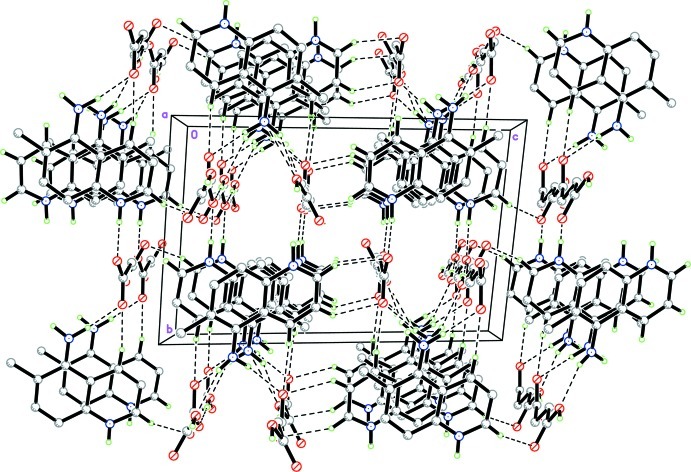
A crystal packing of the title compound, viewed along the a axis. H atoms not involved in the intermolecular interactions (dashed lines) have been omitted for clarity.

## supplementary crystallographic information

### Comment

Recently, hydrogen-bonding patterns involving quinoline and its derivatives with organic acid have been investigated (Thanigaimani *et al.*, 2013*a*,*b*,*c*; Loh *et al.*, 2010). Syntheses of the quinoline derivatives were discussed earlier (Sasaki *et al.*, 1998; Reux *et al.*, 2009). Quinolines and their derivatives are very important compounds because of their wide occurrence in natural products (Morimoto *et al.*, 1991) and biologically active compounds (Markees *et al.*, 1970). Succinic acid derivatives are mostly used in chemicals, food and pharmaceuticals (Sauer *et al.*, 2008). In this paper, we present the X-ray single-crystal structure of 5-amino-6-methylquinolin-1-ium hydrogen succinate (I).

The asymmetric unit of the title salt consists of two crystallographically independent 5-amino-6-methylquinolin-1-ium cations (*A* and *B*) and two 3-carboxypropanoate anions (*A* and *B*) (Fig. 1). Each 5-amino-6-methylquinolin-1-ium cation is essentially planar, with maximum deviations of 0.026 (3) Å for atom C5A in cation *A* and 0.016 (2) Å for C8B atom in cation *B*. In the cations, protonation of atoms N1A and N1B lead to a slight increase in C1A—N1A—C9A [123.1 (2)°] and C1B—N1B—C9B [123.3 (2)°] angles. The bond lengths (Allen *et al.*, 1987) and angles are normal.

In the crystal packing (Fig. 2), the ion units are linked by N1A—H1NA···O4A, N1B—H1NB···O4B, O2A—H10A···O4A^i^, O2B—H10B···O4B^i^, N2A—H2NA···O3A^ii^, N2A—H3NA···O2B^iii^, N2B—H2NB···O2A^ii^ and N2B—H3NB···O3B^ii^ hydrogen bonds (symmetry codes in Table 1), into a three-dimensional network. Furthermore, the crystal structure is stabilized by C7A—H7AA···O3A^ii^, C9A—H9AA···O1A^iv^, C7B—H7BA···O3B^ii^, C8B—H8BA···O3B^v^ and C9B—H9BA···O4B^v^ hydrogen bonds (symmetry codes in Table 1) and π–π stacking interactions between the centroids of C1A–C6A (*Cg*2), N1B/C6B–C9B/C1B (*Cg*4) rings and C1A–C6A, C1B–C6B (C*g*5) rings, with C*g*2···C*g*4 and *Cg*2···*Cg*5 distances of 3.7283 (15) and 3.8467 (15) Å, respectively.

### Experimental

Hot methanol solutions (20 ml) of 5-amino-6-methylquinoline (39 mg, Aldrich) and succinic acid (29 mg, Aldrich) were mixed and warmed over a heating magnetic stirrer hotplate for a few minutes. The resulting solution was allowed to cool slowly at room temperature and crystals of the title compound (I) appeared after a few days.

### Refinement

O- and N-bound H atoms were located in a difference Fourier maps. Atoms H1OB, H2NA, H3NA, H1NB, H2NB and H3NB were refined freely, while atoms H1OA and H1NA were refined with a bond restraint O—H = 0.82 (1) Å and N—H = 0.87 (1) Å [refined distances: O2A—H1OA = 0.834 (10) Å, O2B—H1OB = 0.92 (4) Å, N1A—H1NA = 0.883 (10) Å, N2A—H2NA = 0.98 (5) Å, N2A—H3NA = 0.93 (4) Å, N1B—H1NB = 0.96 (3) Å, N2B—H2NB = 0.87 (3) Å and N2B—H3NB = 0.88 (3) Å]. The remaining H atoms were positioned geometrically (C—H = 0.95–0.99 Å) and were refined using a riding model, with *U*_iso_(H) = 1.2*U*_eq_(C) or 1.5*U*_eq_(methyl C). A rotating-group model was used for the methyl group. Three outliers were omitted (-4 -7 7, -1 -7 12 and -4 -7 6) in the final refinement.

### Crystal data

**Table:**

C_10_H_11_N_2_^+^·C_4_H_5_O_4_^−^	*Z* = 4
*M**_r_* = 276.29	*F*(000) = 584
Triclinic, *P*1	*D*_x_ = 1.357 Mg m^−^^3^
Hall symbol: -P 1	Mo *K*α radiation, λ = 0.71073 Å
*a* = 8.0784 (3) Å	Cell parameters from 5690 reflections
*b* = 10.8234 (4) Å	θ = 2.4–29.7°
*c* = 16.4366 (6) Å	µ = 0.10 mm^−^^1^
α = 91.608 (2)°	*T* = 100 K
β = 101.039 (2)°	Block, orange
γ = 105.782 (2)°	0.31 × 0.17 × 0.16 mm
*V* = 1352.49 (9) Å^3^	

### Data collection

**Table:**

Bruker SMART APEXII CCD area-detector diffractometer	6733 independent reflections
Radiation source: fine-focus sealed tube	4669 reflections with *I* > 2σ(*I*)
Graphite monochromator	*R*_int_ = 0.048
φ and ω scans	θ_max_ = 28.5°, θ_min_ = 1.3°
Absorption correction: multi-scan (*SADABS*; Bruker, 2009)	*h* = −10→10
*T*_min_ = 0.970, *T*_max_ = 0.984	*k* = −14→14
19941 measured reflections	*l* = −21→22

### Refinement

**Table:**

Refinement on *F*^2^	Primary atom site location: structure-invariant direct methods
Least-squares matrix: full	Secondary atom site location: difference Fourier map
*R*[*F*^2^ > 2σ(*F*^2^)] = 0.066	Hydrogen site location: inferred from neighbouring sites
*wR*(*F*^2^) = 0.200	H atoms treated by a mixture of independent and constrained refinement
*S* = 1.04	*w* = 1/[σ^2^(*F*_o_^2^) + (0.0947*P*)^2^ + 1.0362*P*] where *P* = (*F*_o_^2^ + 2*F*_c_^2^)/3
6733 reflections	(Δ/σ)_max_ < 0.001
395 parameters	Δρ_max_ = 0.52 e Å^−^^3^
2 restraints	Δρ_min_ = −0.35 e Å^−^^3^

### Special details

**Table:**

Experimental. The crystal was placed in the cold stream of an Oxford Cryosystems Cobra open-flow nitrogen cryostat (Cosier & Glazer, 1986) operating at 100.0 (1) K.
Geometry. All e.s.d.'s (except the e.s.d. in the dihedral angle between two l.s. planes) are estimated using the full covariance matrix. The cell e.s.d.'s are taken into account individually in the estimation of e.s.d.'s in distances, angles and torsion angles; correlations between e.s.d.'s in cell parameters are only used when they are defined by crystal symmetry. An approximate (isotropic) treatment of cell e.s.d.'s is used for estimating e.s.d.'s involving l.s. planes.
Refinement. Refinement of *F*^2^ against ALL reflections. The weighted *R*-factor *wR* and goodness of fit *S* are based on *F*^2^, conventional *R*-factors *R* are based on *F*, with *F* set to zero for negative *F*^2^. The threshold expression of *F*^2^ > σ(*F*^2^) is used only for calculating *R*-factors(gt) *etc*. and is not relevant to the choice of reflections for refinement. *R*-factors based on *F*^2^ are statistically about twice as large as those based on *F*, and *R*-factors based on ALL data will be even larger.

### Fractional atomic coordinates and isotropic or equivalent isotropic displacement parameters (Å^2^)

**Table:**

	*x*	*y*	*z*	*U*_iso_*/*U*_eq_	
N1A	0.1452 (3)	0.6288 (2)	0.13378 (13)	0.0263 (4)	
N2A	0.1161 (3)	1.0273 (2)	0.25781 (16)	0.0356 (5)	
C1A	0.1331 (3)	0.6995 (2)	0.20182 (15)	0.0259 (5)	
C2A	0.1184 (3)	0.6413 (3)	0.27674 (16)	0.0299 (6)	
H2AA	0.1176	0.5538	0.2815	0.036*	
C3A	0.1054 (4)	0.7167 (3)	0.34280 (17)	0.0339 (6)	
H3AA	0.0964	0.6792	0.3939	0.041*	
C4A	0.1047 (3)	0.8441 (3)	0.33871 (16)	0.0318 (6)	
C5A	0.1197 (3)	0.9047 (2)	0.26446 (16)	0.0282 (5)	
C6A	0.1369 (3)	0.8304 (2)	0.19393 (15)	0.0248 (5)	
C7A	0.1584 (3)	0.8819 (3)	0.11766 (16)	0.0281 (5)	
H7AA	0.1630	0.9696	0.1113	0.034*	
C8A	0.1728 (4)	0.8061 (3)	0.05216 (16)	0.0325 (6)	
H8AA	0.1886	0.8414	0.0010	0.039*	
C9A	0.1641 (3)	0.6781 (3)	0.06169 (17)	0.0318 (6)	
H9AA	0.1717	0.6249	0.0164	0.038*	
C10A	0.0868 (5)	0.9222 (3)	0.41296 (18)	0.0463 (8)	
H10A	0.0772	0.8685	0.4598	0.069*	
H10B	−0.0189	0.9515	0.3984	0.069*	
H10C	0.1905	0.9970	0.4287	0.069*	
O1A	0.7074 (2)	0.44158 (16)	0.09215 (11)	0.0275 (4)	
O2A	0.7631 (2)	0.26649 (17)	0.14664 (12)	0.0279 (4)	
O3A	0.1102 (2)	0.17893 (16)	0.11289 (12)	0.0294 (4)	
O4A	0.0728 (2)	0.37301 (16)	0.13103 (12)	0.0273 (4)	
C11A	0.6595 (3)	0.3411 (2)	0.12228 (14)	0.0217 (5)	
C12A	0.4757 (3)	0.2834 (2)	0.13614 (15)	0.0228 (5)	
H12A	0.4818	0.2735	0.1962	0.027*	
H12B	0.4278	0.1964	0.1063	0.027*	
C13A	0.3505 (3)	0.3625 (2)	0.10726 (16)	0.0253 (5)	
H13A	0.3478	0.3762	0.0478	0.030*	
H13B	0.3941	0.4480	0.1392	0.030*	
C14A	0.1656 (3)	0.2977 (2)	0.11840 (15)	0.0228 (5)	
N1B	0.5783 (3)	0.6411 (2)	0.37371 (12)	0.0233 (4)	
N2B	0.6094 (3)	1.0271 (2)	0.22984 (14)	0.0264 (5)	
C1B	0.6078 (3)	0.7071 (2)	0.30499 (15)	0.0219 (5)	
C2B	0.6486 (3)	0.6475 (2)	0.23808 (15)	0.0246 (5)	
H2BA	0.6568	0.5616	0.2390	0.029*	
C3B	0.6768 (3)	0.7175 (2)	0.17047 (15)	0.0257 (5)	
H3BA	0.7049	0.6779	0.1247	0.031*	
C4B	0.6658 (3)	0.8439 (2)	0.16653 (15)	0.0228 (5)	
C5B	0.6250 (3)	0.9052 (2)	0.23322 (14)	0.0214 (5)	
C6B	0.5952 (3)	0.8359 (2)	0.30487 (14)	0.0206 (5)	
C7B	0.5578 (3)	0.8897 (2)	0.37612 (15)	0.0237 (5)	
H7BA	0.5508	0.9759	0.3778	0.028*	
C8B	0.5312 (3)	0.8191 (2)	0.44323 (15)	0.0263 (5)	
H8BA	0.5066	0.8561	0.4910	0.032*	
C9B	0.5410 (3)	0.6919 (2)	0.43990 (15)	0.0254 (5)	
H9BA	0.5209	0.6416	0.4854	0.031*	
C10B	0.7013 (4)	0.9169 (2)	0.09179 (16)	0.0299 (6)	
H10D	0.7268	0.8611	0.0508	0.045*	
H10E	0.8024	0.9931	0.1092	0.045*	
H10F	0.5977	0.9438	0.0670	0.045*	
O1B	1.2074 (2)	0.44971 (16)	0.43052 (11)	0.0258 (4)	
O2B	1.2173 (2)	0.27375 (18)	0.35962 (13)	0.0333 (4)	
O3B	0.5817 (2)	0.19433 (15)	0.37244 (11)	0.0264 (4)	
O4B	0.5389 (2)	0.38843 (15)	0.37072 (11)	0.0248 (4)	
C11B	1.1346 (3)	0.3473 (2)	0.39056 (14)	0.0220 (5)	
C12B	0.9375 (3)	0.2871 (2)	0.36983 (16)	0.0255 (5)	
H12C	0.9079	0.2113	0.4022	0.031*	
H12D	0.9010	0.2562	0.3101	0.031*	
C13B	0.8340 (3)	0.3788 (2)	0.38803 (16)	0.0232 (5)	
H13C	0.8529	0.4497	0.3511	0.028*	
H13D	0.8790	0.4170	0.4462	0.028*	
C14B	0.6382 (3)	0.3128 (2)	0.37564 (14)	0.0199 (4)	
H1OA	0.8673 (18)	0.301 (3)	0.144 (2)	0.041 (9)*	
H1OB	1.335 (5)	0.317 (3)	0.364 (2)	0.053 (10)*	
H1NA	0.125 (4)	0.5444 (11)	0.134 (2)	0.041 (9)*	
H2NA	0.136 (6)	1.074 (4)	0.208 (3)	0.082 (14)*	
H3NA	0.127 (5)	1.084 (4)	0.303 (2)	0.061 (11)*	
H1NB	0.574 (4)	0.551 (3)	0.369 (2)	0.053 (10)*	
H2NB	0.660 (4)	1.079 (3)	0.197 (2)	0.036 (8)*	
H3NB	0.593 (4)	1.069 (3)	0.272 (2)	0.039 (9)*	

### Atomic displacement parameters (Å^2^)

**Table:**

	*U*^11^	*U*^22^	*U*^33^	*U*^12^	*U*^13^	*U*^23^
N1A	0.0259 (11)	0.0269 (11)	0.0281 (11)	0.0100 (9)	0.0065 (9)	0.0043 (9)
N2A	0.0424 (14)	0.0305 (12)	0.0329 (13)	0.0111 (10)	0.0050 (11)	−0.0046 (10)
C1A	0.0206 (12)	0.0312 (13)	0.0244 (12)	0.0068 (10)	0.0019 (9)	0.0008 (10)
C2A	0.0267 (13)	0.0321 (13)	0.0295 (13)	0.0065 (11)	0.0047 (10)	0.0036 (10)
C3A	0.0348 (15)	0.0394 (15)	0.0244 (13)	0.0082 (12)	0.0018 (11)	0.0051 (11)
C4A	0.0261 (13)	0.0426 (15)	0.0227 (12)	0.0062 (11)	0.0014 (10)	−0.0046 (11)
C5A	0.0234 (12)	0.0295 (13)	0.0276 (13)	0.0039 (10)	0.0015 (10)	−0.0025 (10)
C6A	0.0186 (11)	0.0321 (13)	0.0216 (12)	0.0059 (9)	0.0010 (9)	0.0014 (9)
C7A	0.0250 (12)	0.0325 (13)	0.0268 (13)	0.0095 (10)	0.0038 (10)	0.0033 (10)
C8A	0.0317 (14)	0.0441 (15)	0.0229 (12)	0.0119 (12)	0.0069 (10)	0.0043 (11)
C9A	0.0310 (14)	0.0399 (15)	0.0264 (13)	0.0126 (12)	0.0071 (11)	−0.0008 (11)
C10A	0.062 (2)	0.0485 (18)	0.0275 (15)	0.0143 (16)	0.0089 (14)	−0.0036 (13)
O1A	0.0267 (9)	0.0261 (9)	0.0340 (10)	0.0112 (7)	0.0106 (8)	0.0074 (7)
O2A	0.0184 (9)	0.0324 (10)	0.0374 (10)	0.0120 (7)	0.0080 (7)	0.0139 (8)
O3A	0.0216 (9)	0.0238 (9)	0.0424 (11)	0.0076 (7)	0.0035 (8)	0.0056 (7)
O4A	0.0204 (8)	0.0232 (8)	0.0414 (11)	0.0097 (7)	0.0083 (7)	0.0048 (7)
C11A	0.0209 (11)	0.0252 (12)	0.0200 (11)	0.0081 (9)	0.0049 (9)	−0.0003 (9)
C12A	0.0202 (11)	0.0248 (11)	0.0270 (12)	0.0113 (9)	0.0053 (9)	0.0078 (9)
C13A	0.0195 (11)	0.0241 (12)	0.0324 (13)	0.0062 (9)	0.0052 (10)	0.0035 (10)
C14A	0.0181 (11)	0.0256 (12)	0.0235 (11)	0.0068 (9)	0.0003 (9)	0.0035 (9)
N1B	0.0232 (10)	0.0232 (10)	0.0229 (10)	0.0078 (8)	0.0014 (8)	0.0043 (8)
N2B	0.0323 (12)	0.0225 (10)	0.0264 (11)	0.0090 (9)	0.0086 (9)	0.0059 (9)
C1B	0.0187 (11)	0.0223 (11)	0.0232 (12)	0.0071 (9)	−0.0014 (9)	0.0002 (9)
C2B	0.0269 (12)	0.0225 (11)	0.0258 (12)	0.0113 (10)	0.0027 (10)	0.0017 (9)
C3B	0.0261 (12)	0.0282 (12)	0.0235 (12)	0.0118 (10)	0.0016 (10)	−0.0021 (9)
C4B	0.0217 (11)	0.0243 (11)	0.0209 (11)	0.0052 (9)	0.0026 (9)	0.0021 (9)
C5B	0.0202 (11)	0.0208 (11)	0.0227 (11)	0.0079 (9)	0.0001 (9)	0.0003 (9)
C6B	0.0190 (11)	0.0193 (11)	0.0220 (11)	0.0055 (8)	0.0007 (9)	0.0014 (8)
C7B	0.0241 (12)	0.0219 (11)	0.0251 (12)	0.0069 (9)	0.0044 (9)	0.0025 (9)
C8B	0.0284 (13)	0.0277 (12)	0.0227 (12)	0.0075 (10)	0.0058 (10)	0.0005 (9)
C9B	0.0236 (12)	0.0302 (13)	0.0219 (12)	0.0071 (10)	0.0036 (9)	0.0041 (9)
C10B	0.0342 (14)	0.0303 (13)	0.0252 (13)	0.0085 (11)	0.0074 (11)	0.0025 (10)
O1B	0.0215 (8)	0.0259 (9)	0.0287 (9)	0.0072 (7)	0.0021 (7)	−0.0024 (7)
O2B	0.0185 (9)	0.0301 (9)	0.0496 (12)	0.0073 (8)	0.0051 (8)	−0.0128 (8)
O3B	0.0235 (9)	0.0204 (8)	0.0380 (10)	0.0077 (7)	0.0105 (7)	0.0026 (7)
O4B	0.0193 (8)	0.0234 (8)	0.0336 (10)	0.0098 (7)	0.0049 (7)	0.0025 (7)
C11B	0.0212 (11)	0.0254 (11)	0.0218 (11)	0.0119 (9)	0.0022 (9)	0.0028 (9)
C12B	0.0202 (11)	0.0262 (12)	0.0303 (13)	0.0095 (9)	0.0023 (9)	−0.0028 (10)
C13B	0.0191 (11)	0.0220 (11)	0.0315 (13)	0.0100 (9)	0.0061 (9)	0.0033 (9)
C14B	0.0218 (11)	0.0228 (11)	0.0177 (10)	0.0099 (9)	0.0050 (9)	0.0021 (8)

### Geometric parameters (Å, º)

**Table:**

N1A—C9A	1.331 (3)	N1B—C9B	1.326 (3)
N1A—C1A	1.371 (3)	N1B—C1B	1.378 (3)
N1A—H1NA	0.883 (10)	N1B—H1NB	0.96 (3)
N2A—C5A	1.342 (3)	N2B—C5B	1.361 (3)
N2A—H2NA	0.98 (5)	N2B—H2NB	0.87 (3)
N2A—H3NA	0.93 (4)	N2B—H3NB	0.88 (3)
C1A—C2A	1.409 (4)	C1B—C2B	1.396 (3)
C1A—C6A	1.419 (3)	C1B—C6B	1.426 (3)
C2A—C3A	1.377 (4)	C2B—C3B	1.382 (3)
C2A—H2AA	0.9500	C2B—H2BA	0.9500
C3A—C4A	1.384 (4)	C3B—C4B	1.398 (3)
C3A—H3AA	0.9500	C3B—H3BA	0.9500
C4A—C5A	1.412 (4)	C4B—C5B	1.402 (3)
C4A—C10A	1.517 (4)	C4B—C10B	1.510 (3)
C5A—C6A	1.442 (3)	C5B—C6B	1.437 (3)
C6A—C7A	1.408 (3)	C6B—C7B	1.412 (3)
C7A—C8A	1.377 (4)	C7B—C8B	1.377 (3)
C7A—H7AA	0.9500	C7B—H7BA	0.9500
C8A—C9A	1.383 (4)	C8B—C9B	1.401 (3)
C8A—H8AA	0.9500	C8B—H8BA	0.9500
C9A—H9AA	0.9500	C9B—H9BA	0.9500
C10A—H10A	0.9800	C10B—H10D	0.9800
C10A—H10B	0.9800	C10B—H10E	0.9800
C10A—H10C	0.9800	C10B—H10F	0.9800
O1A—C11A	1.205 (3)	O1B—C11B	1.209 (3)
O2A—C11A	1.331 (3)	O2B—C11B	1.317 (3)
O2A—H1OA	0.834 (10)	O2B—H1OB	0.92 (4)
O3A—C14A	1.235 (3)	O3B—C14B	1.235 (3)
O4A—C14A	1.285 (3)	O4B—C14B	1.286 (3)
C11A—C12A	1.510 (3)	C11B—C12B	1.514 (3)
C12A—C13A	1.514 (3)	C12B—C13B	1.518 (3)
C12A—H12A	0.9900	C12B—H12C	0.9900
C12A—H12B	0.9900	C12B—H12D	0.9900
C13A—C14A	1.516 (3)	C13B—C14B	1.519 (3)
C13A—H13A	0.9900	C13B—H13C	0.9900
C13A—H13B	0.9900	C13B—H13D	0.9900
			
C9A—N1A—C1A	123.1 (2)	C9B—N1B—C1B	123.3 (2)
C9A—N1A—H1NA	116 (2)	C9B—N1B—H1NB	121 (2)
C1A—N1A—H1NA	120 (2)	C1B—N1B—H1NB	116 (2)
C5A—N2A—H2NA	123 (2)	C5B—N2B—H2NB	121 (2)
C5A—N2A—H3NA	123 (2)	C5B—N2B—H3NB	122 (2)
H2NA—N2A—H3NA	111 (3)	H2NB—N2B—H3NB	112 (3)
N1A—C1A—C2A	119.7 (2)	N1B—C1B—C2B	120.0 (2)
N1A—C1A—C6A	118.1 (2)	N1B—C1B—C6B	118.1 (2)
C2A—C1A—C6A	122.2 (2)	C2B—C1B—C6B	121.9 (2)
C3A—C2A—C1A	117.1 (2)	C3B—C2B—C1B	117.9 (2)
C3A—C2A—H2AA	121.5	C3B—C2B—H2BA	121.1
C1A—C2A—H2AA	121.5	C1B—C2B—H2BA	121.1
C2A—C3A—C4A	123.5 (3)	C2B—C3B—C4B	122.9 (2)
C2A—C3A—H3AA	118.2	C2B—C3B—H3BA	118.5
C4A—C3A—H3AA	118.2	C4B—C3B—H3BA	118.5
C3A—C4A—C5A	120.5 (2)	C3B—C4B—C5B	119.9 (2)
C3A—C4A—C10A	121.4 (3)	C3B—C4B—C10B	120.7 (2)
C5A—C4A—C10A	118.1 (3)	C5B—C4B—C10B	119.4 (2)
N2A—C5A—C4A	122.1 (2)	N2B—C5B—C4B	120.9 (2)
N2A—C5A—C6A	119.9 (2)	N2B—C5B—C6B	120.2 (2)
C4A—C5A—C6A	118.1 (2)	C4B—C5B—C6B	118.9 (2)
C7A—C6A—C1A	118.4 (2)	C7B—C6B—C1B	118.1 (2)
C7A—C6A—C5A	123.1 (2)	C7B—C6B—C5B	123.5 (2)
C1A—C6A—C5A	118.6 (2)	C1B—C6B—C5B	118.4 (2)
C8A—C7A—C6A	120.6 (2)	C8B—C7B—C6B	121.0 (2)
C8A—C7A—H7AA	119.7	C8B—C7B—H7BA	119.5
C6A—C7A—H7AA	119.7	C6B—C7B—H7BA	119.5
C7A—C8A—C9A	119.3 (2)	C7B—C8B—C9B	118.9 (2)
C7A—C8A—H8AA	120.4	C7B—C8B—H8BA	120.5
C9A—C8A—H8AA	120.4	C9B—C8B—H8BA	120.5
N1A—C9A—C8A	120.6 (2)	N1B—C9B—C8B	120.5 (2)
N1A—C9A—H9AA	119.7	N1B—C9B—H9BA	119.8
C8A—C9A—H9AA	119.7	C8B—C9B—H9BA	119.8
C4A—C10A—H10A	109.5	C4B—C10B—H10D	109.5
C4A—C10A—H10B	109.5	C4B—C10B—H10E	109.5
H10A—C10A—H10B	109.5	H10D—C10B—H10E	109.5
C4A—C10A—H10C	109.5	C4B—C10B—H10F	109.5
H10A—C10A—H10C	109.5	H10D—C10B—H10F	109.5
H10B—C10A—H10C	109.5	H10E—C10B—H10F	109.5
C11A—O2A—H1OA	112 (2)	C11B—O2B—H1OB	111 (2)
O1A—C11A—O2A	123.7 (2)	O1B—C11B—O2B	124.1 (2)
O1A—C11A—C12A	124.7 (2)	O1B—C11B—C12B	124.5 (2)
O2A—C11A—C12A	111.64 (19)	O2B—C11B—C12B	111.4 (2)
C11A—C12A—C13A	113.97 (19)	C11B—C12B—C13B	113.5 (2)
C11A—C12A—H12A	108.8	C11B—C12B—H12C	108.9
C13A—C12A—H12A	108.8	C13B—C12B—H12C	108.9
C11A—C12A—H12B	108.8	C11B—C12B—H12D	108.9
C13A—C12A—H12B	108.8	C13B—C12B—H12D	108.9
H12A—C12A—H12B	107.7	H12C—C12B—H12D	107.7
C12A—C13A—C14A	112.12 (19)	C12B—C13B—C14B	112.63 (19)
C12A—C13A—H13A	109.2	C12B—C13B—H13C	109.1
C14A—C13A—H13A	109.2	C14B—C13B—H13C	109.1
C12A—C13A—H13B	109.2	C12B—C13B—H13D	109.1
C14A—C13A—H13B	109.2	C14B—C13B—H13D	109.1
H13A—C13A—H13B	107.9	H13C—C13B—H13D	107.8
O3A—C14A—O4A	123.7 (2)	O3B—C14B—O4B	123.4 (2)
O3A—C14A—C13A	120.2 (2)	O3B—C14B—C13B	121.0 (2)
O4A—C14A—C13A	116.2 (2)	O4B—C14B—C13B	115.60 (19)
			
C9A—N1A—C1A—C2A	−178.1 (2)	C9B—N1B—C1B—C2B	179.2 (2)
C9A—N1A—C1A—C6A	1.5 (4)	C9B—N1B—C1B—C6B	−0.8 (3)
N1A—C1A—C2A—C3A	−179.5 (2)	N1B—C1B—C2B—C3B	179.9 (2)
C6A—C1A—C2A—C3A	0.9 (4)	C6B—C1B—C2B—C3B	−0.1 (3)
C1A—C2A—C3A—C4A	0.5 (4)	C1B—C2B—C3B—C4B	−0.1 (4)
C2A—C3A—C4A—C5A	−0.7 (4)	C2B—C3B—C4B—C5B	0.1 (4)
C2A—C3A—C4A—C10A	178.9 (3)	C2B—C3B—C4B—C10B	178.8 (2)
C3A—C4A—C5A—N2A	178.7 (3)	C3B—C4B—C5B—N2B	−178.4 (2)
C10A—C4A—C5A—N2A	−0.9 (4)	C10B—C4B—C5B—N2B	2.9 (4)
C3A—C4A—C5A—C6A	−0.5 (4)	C3B—C4B—C5B—C6B	0.1 (3)
C10A—C4A—C5A—C6A	179.9 (2)	C10B—C4B—C5B—C6B	−178.6 (2)
N1A—C1A—C6A—C7A	−1.9 (3)	N1B—C1B—C6B—C7B	1.6 (3)
C2A—C1A—C6A—C7A	177.7 (2)	C2B—C1B—C6B—C7B	−178.4 (2)
N1A—C1A—C6A—C5A	178.4 (2)	N1B—C1B—C6B—C5B	−179.7 (2)
C2A—C1A—C6A—C5A	−2.0 (4)	C2B—C1B—C6B—C5B	0.3 (3)
N2A—C5A—C6A—C7A	2.9 (4)	N2B—C5B—C6B—C7B	−3.2 (4)
C4A—C5A—C6A—C7A	−177.9 (2)	C4B—C5B—C6B—C7B	178.3 (2)
N2A—C5A—C6A—C1A	−177.5 (2)	N2B—C5B—C6B—C1B	178.2 (2)
C4A—C5A—C6A—C1A	1.7 (3)	C4B—C5B—C6B—C1B	−0.3 (3)
C1A—C6A—C7A—C8A	0.9 (4)	C1B—C6B—C7B—C8B	−1.1 (3)
C5A—C6A—C7A—C8A	−179.5 (2)	C5B—C6B—C7B—C8B	−179.7 (2)
C6A—C7A—C8A—C9A	0.7 (4)	C6B—C7B—C8B—C9B	−0.3 (4)
C1A—N1A—C9A—C8A	0.1 (4)	C1B—N1B—C9B—C8B	−0.6 (4)
C7A—C8A—C9A—N1A	−1.2 (4)	C7B—C8B—C9B—N1B	1.1 (4)
O1A—C11A—C12A—C13A	0.9 (3)	O1B—C11B—C12B—C13B	12.1 (3)
O2A—C11A—C12A—C13A	−178.4 (2)	O2B—C11B—C12B—C13B	−168.0 (2)
C11A—C12A—C13A—C14A	177.1 (2)	C11B—C12B—C13B—C14B	−173.8 (2)
C12A—C13A—C14A—O3A	−31.2 (3)	C12B—C13B—C14B—O3B	17.2 (3)
C12A—C13A—C14A—O4A	150.7 (2)	C12B—C13B—C14B—O4B	−164.3 (2)

### Hydrogen-bond geometry (Å, º)

**Table:**

*D*—H···*A*	*D*—H	H···*A*	*D*···*A*	*D*—H···*A*
N1*A*—H1*NA*···O4*A*	0.88 (1)	1.78 (1)	2.667 (3)	177 (4)
N1*B*—H1*NB*···O4*B*	0.97 (3)	1.71 (3)	2.664 (3)	170 (3)
O2*A*—H1*OA*···O4*A*^i^	0.83 (2)	1.69 (2)	2.520 (2)	176 (3)
O2*B*—H1*OB*···O4*B*^i^	0.93 (4)	1.60 (4)	2.525 (2)	179 (4)
N2*A*—H2*NA*···O3*A*^ii^	0.99 (5)	1.97 (5)	2.931 (3)	163 (4)
N2*A*—H3*NA*···O2*B*^iii^	0.93 (4)	2.11 (4)	2.937 (3)	149 (3)
N2*B*—H2*NB*···O2*A*^ii^	0.87 (3)	2.22 (3)	3.037 (3)	157 (3)
N2*B*—H3*NB*···O3*B*^ii^	0.87 (3)	2.14 (3)	3.001 (3)	172 (3)
C7*A*—H7*AA*···O3*A*^ii^	0.95	2.42	3.343 (3)	165
C9*A*—H9*AA*···O1*A*^iv^	0.95	2.37	3.271 (3)	158
C7*B*—H7*BA*···O3*B*^ii^	0.95	2.31	3.253 (3)	169
C8*B*—H8*BA*···O3*B*^v^	0.95	2.51	3.323 (3)	143
C9*B*—H9*BA*···O4*B*^v^	0.95	2.52	3.388 (3)	153

Symmetry codes: (i) *x*+1, *y*, *z*; (ii) *x*, *y*+1, *z*; (iii) *x*−1, *y*+1, *z*; (iv) −*x*+1, −*y*+1, −*z*; (v) −*x*+1, −*y*+1, −*z*+1.
